# Evaluation of Salivary Cytokines and Vitamin D Levels in Periodontopathic Patients

**DOI:** 10.3390/ijms21082669

**Published:** 2020-04-11

**Authors:** Erica Costantini, Bruna Sinjari, Francesca Piscopo, Annamaria Porreca, Marcella Reale, Sergio Caputi, Giovanna Murmura

**Affiliations:** 1 Department of Medical, Oral and Biotechnological Sciences, University “G. d’Annunzio” Chieti-Pescara, 66100 Chieti, Italy; erica.costantini@unich.it (E.C.); b.sinjari@unich.it (B.S.); frapis.fp93@gmail.com (F.P.); scaputi@unich.it (S.C.); gmurmura@unich.it (G.M.); 2 Department of Economic Studies, University G. d’Annunzio Chieti–Pescara, 65100 Pescara, Italy; annamaria.porreca@unich.it

**Keywords:** cytokines, vitamin D, inflammation, saliva, periodontal disease

## Abstract

Periodontal disease (PD) is an inflammatory condition of the tissues supporting the teeth, which is widespread among the adult population. Evidence shows a relationship between PD and vitamin D levels, which is involved in the regulation of bone metabolism, mineral homeostasis, and inflammatory response. This study aimed to perform a simultaneous evaluation of inflammatory mediators and vitamin D levels in saliva in periodontopathic patients to better understand their role in periodontal disease. In this observational study, clinical periodontal parameter examination was performed for each patient. Moreover, the saliva levels of 25(OH)D_3_, TGFβ, IL-35, IL-17A, and MMP9 were evaluated using an ELISA assay. An increase in TGFβ, IL-35, MMP9, and IL-17A salivary levels and a reduction in 25(OH)D_3_ levels were observed in periodontopathic patients with respect to the healthy controls. The present study revealed significant positive correlation between cytokines and highly negative correlation between 25(OH)D_3_ and salivary cytokine levels. Further studies are needed to better understand if salivary cytokines and vitamin D evaluation may represent a new approach for detection and prevention of progressive diseases, such as PD.

## 1. Introduction

Periodontal disease (PD) is an inflammatory condition affecting the periodontium, including the gingiva, cementum, periodontal ligament, and alveolar bone, representing tooth-supporting structures. It is estimated that 10–15% of the world’s population is affected by PD [1] due to a complex interaction between genetic and epigenetic factors, such as age, sex, smoking, and systemic health [2,3]. Even though many studies suggest a primary role for bacteria in the etiology of destructive periodontal disease [4], it is nowadays possible to assert that periodontitis is initiated by an unbalanced interaction between the oral microbial community and the host inflammatory response to the microbial challenges. Such dysregulated immune–inflammatory processes are responsible for the majority of host tissue destruction, leading to tooth loss [5]. Periodontitis is associated with imbalanced immune homeostasis in the oral mucosa, with increased bacterial growth and multiplication in the dental plaque. Most of these microorganisms induce an increase in pH and a reduction in the local redox potential, with the upregulation of inflammatory mediators [6] leading to the onset and progression of the disease [7,8]. Immune response in PD is imbalanced, with 70% of B-cell subset dominance and only 30% of the macrophage, neutrophil, and T-cell subset [9,10], in association with an increased level of inflammatory cytokines like interleukin (IL)-1, tumor necrosis factor (TNF)-α, interferon (IFN)-γ, IL-17, and IL-6 [9,11]. Emerging evidence suggests that the promotion of proinflammatory cytokine secretion is referred to the activation of intracellular protein complex, inflammasome, which plays a key role in the pathogenesis of periodontitis [12]. Moreover, previous studies have shown strong correlation between vitamin D and oral health through its role in the inhibition of complex nod-like receptor leucine-rich repeat protein 3 (NLRP3) inflammasome activation via vitamin D receptor (VDR) signaling in bone metabolism, immune functions, and antimicrobial peptide production [12,13].

Vitamin D is a nutrient that is gaining increased importance for its essential role in calcium and bone metabolism and homeostasis [14,15]. Mineralization and maintenance of tissue integrity [6,16] are the main functions of vitamin D and are necessary for oral health preservation. Recently, various studies have demonstrated that the average vitamin D intake is 10 μg/day, indicating vitamin D deficiency in a surprisingly large part of the population [13,17].

The circulating metabolite of vitamin D, 25-hydroxy vitamin D_3_ (25(OH)D_3_), binds the vitamin D receptor [18], playing an important role in oral health maintenance through antibacterial, antiviral, and anti-inflammatory activity. The active form of vitamin D (1,25(OH)2D_3_) leads to the regulation of both innate and adaptive immunity, modulating inflammatory cytokine production and blocking antigen-presenting dendritic cell maturation with a decrease of antigen-specific T-cell activation and proliferation, associated with regulatory T cell (Treg) activity promotion [7,18,19]. Liu et al. [20] have demonstrated the involvement of 25(OH)D_3_ and IL-1β in patients with generalized aggressive periodontitis, both in local and systemic evaluation. Inflammation and destruction of the tooth support structure in periodontal tissues may be related to pro- and anti-inflammatory mediators. Indeed, infiltrated plasma cells and proinflammatory cytokine production in PD patients potentially regulate bone homeostasis and calcium imbalance, associated with low bone mass, alveolar bone resorption, and tooth loss, which are the main features of periodontitis.

Bone loss and overplayed immune response to bacterial invasion are the major events in periodontitis pathogenesis; thus, lack of vitamin D may be a causal factor for PD development and progression. However, conflicting results on the correlation between vitamin D deficiency and PD onset have been reported, considering the involvement of multiple endogenous and exogenous factors in the development of periodontitis [21].

Saliva, which may be a mirror of the body, has been considered as a new investigative tool to monitor general health and the onset of specific diseases. Saliva is a complex combination of water and organic and inorganic components like enzymes, mucinous substances, and antibacterial components. It is in contact with the teeth and oral mucosa, reflecting and regulating oral cavity homeostasis through lubrication, pH regulation, and antibacterial and immune activity [22,23]. Being easily accessible and due to the variety of secreted components, saliva can be used for immunological and nutritional evaluation. Thus, researchers are currently investigating the possible use of saliva for disease assessment and identification of biomarkers that can act as early sentinels of disease. Several studies have suggested that inflammatory response during periodontitis may mediate some dangerous crosstalk between oral and extraoral sites and evaluate the presence of metabolites and immunological components in saliva and serum [22,23,24]. The study by Bahramian et al. showed a significant positive correlation between serum and salivary levels of vitamin D in patients with recurrent aphthous stomatitis compared to healthy subjects [22].

Taking into account that vitamin D3 modulates cytokines expression by acting in an autocrine/paracrine manner on human gingival fibroblasts (hGF) and human periodontal ligament cells (hPDLc) [25,26,27], which increases also the differentiation of keratinocytes [28], the aim of this study was the evaluation of Th1- and Th17-related cytokines, along with transforming growth factor (TGF) β and matrix metalloproteinase (MMP)9, and 25(OH)D_3_ in periodontitis to understand their involvement in inflammatory reaction in the oral cavity.

## 2. Results

### 2.1. Clinical Findings and Periodontal Parameters

Clinical periodontal measurements of the subjects in the study groups are outlined in Table 1. Probing depth and clinical attachment level (CAL) are the most important clinical parameters in the assessment, diagnosis, and management of periodontal disease. The mean values and standard deviation of CAL (CAL 12–21, 31–41, 36, 46, 26, and 16), maximal probing depth, full-mouth plaque score (FMPS), full-mouth bleeding score (FMBS), and radiographic bone loss in the periodontitis groups are reported and relate to disease stage.

### 2.2. Salivary Levels of Inflammatory Markers and Vitamin D

The measurement of inflammatory markers in the saliva was considered for the entire study population, independently of periodontal disease status. Salivary TGFβ, IL-35, and IL-17A levels were significantly higher in the PD group (*p* < 0.001) than those in the healthy control (HC) group. However, there were no significant differences in MMP9 between the PD and HC groups (*p* = 0.2), although levels were higher in PD patients (Table 2). With regard to vitamin D, the PD group showed lower levels than those of the HC group but without significant differences (*p* = 0.207), as reported in Table 2.

### 2.3. Correlation between Salivary Parameters

Table 3 shows the Spearman rho correlation obtained from salivary marker levels in the PD and HC groups. Data revealed strong negative correlation between 25(OH)D_3_ and TGFβ levels (*p* = 0.01), IL-35 (*p* < 0.001), and IL-17A (*p* < 0.05), suggesting that low levels of 25(OH)D_3_ could increase inflammatory response. An inverse correlation was found between MMP9 and 25(OH)D_3_, although it was not significant. Significant positive correlation (*p* < 0.05) was also observed between cytokines, mostly with TGFβ levels. Moreover, the data revealed inverse correlation between 25(OH)D_3_ and TGFβ levels (*p* = 0.01) and IL-17A (*p* < 0.05). IL-35 and MMP9 showed negative correlation with 25(OH)D_3_, while a positive correlation (*p* < 0.05) was observed between TGFβ, IL-17A, and MMP9.

### 2.4. Correlation between Salivary Parameters and Disease Stage

**Figure 1:**
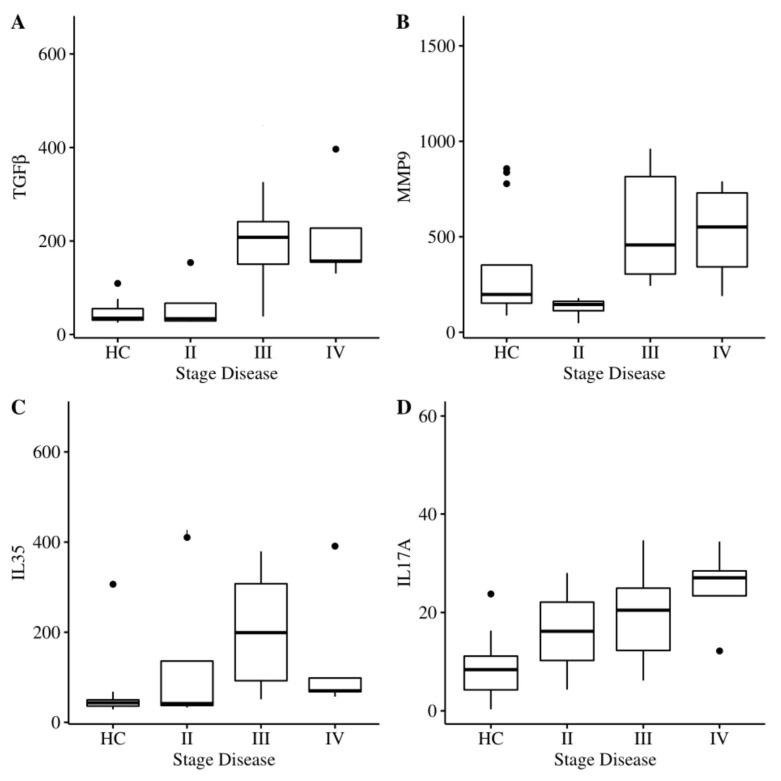
Box–whisker graphs of TGFβ (panel **A**), MMP9 (panel **B**), IL-35 (panel **C**), and IL-17A (panel **D**) in the HC and patients in three stages of disease. Box–whisker plots show 25th and 75th percentile range (box) with 95% confidence interval (whiskers) and median values (transverse lines in box).

To investigate the possible association between inflammatory markers and disease severity, the study population was divided according to clinical parameters and compared with the HC. Figure 1A–D represents the boxplots of the median and interquartile range (IQR) of periodontopathic patients divided into three different stages of the disease and of the HC. As shown in panel A, salivary TGFβ levels showed an upward trend with disease stage, suggesting a possible role of TGFβ in the induction of new extracellular matrix (ECM) component synthesis. MMP9, as reported in panel B, showed a rising trend, supporting the proinflammatory role of metalloproteinase in PD. IL-35 levels followed a different trend compared to the other cytokines. We observed increasing IL-35 levels in stages II and III compared to HC, while a decline of IL-35 was observed in stage IV compared to stages II and III, confirming the imbalance between pro- and anti-inflammatory cytokines in favor of inflammatory ones. Panel D indicates that IL-17A, the main proinflammatory cytokine involved in PD, showed an increase with stage. However, we observed a decline of 25(OH)D_3_ levels in stage IV with respect to stages II and III, which, although higher than that in HC (data not shown), may be due to the low sample size.

## 3. Discussion

Periodontitis is a common form of oral pathology, defining a chronic inflammatory disorder with the destruction of tooth-supporting tissue and bone [29].

In this preliminary study, we aimed to better understand periodontopathic patient evaluation, especially from a perspective of inflammatory markers and vitamin D assessment, through an ELISA assay. The evaluation of vitamin D levels is commonly performed in serum, and only a few studies have assessed its levels in gingival crevicular fluid (GCF), which is considered as serum exudate [30,31]. Both blood and GCF sampling have relative invasiveness linked to their withdrawal method.

Saliva, due to its easy access and noninvasiveness, may be a very attractive fluid to follow the course of the disease for detection of immune–pathological changes or treatment follow-up in systemic diseases [32]. Due to its easy collection without any specially trained professional requirements, we selected saliva as the biological fluid to evaluate levels of vitamin D and cytokines.

In the present study, we observed a significant increase in TGFβ, IL-35, MMP9, and IL-17A levels in PD patients when compared to HC, supporting the presence of an inflammatory microenvironment.

In fact, TGFβ is a pleiotropic cytokine acting in cell proliferation, differentiation, chemotaxis, and apoptosis, exhibiting both pro- and anti-inflammatory properties [33,34]. Besides the ability of TGFβ to stimulate the migration and synthesis of ECM molecules and to inhibit ECM breakdown, it has been intensively evaluated in relation to all types of gingival enlargement as well as tooth development and reparative processes [33,35]. Therefore, high salivary TGFβ levels in patients with periodontitis may be a modulator of inflammatory response against persistent bacterial aggression or may minimize the destruction of supporting tooth tissue, thus modulating the progression of periodontitis [36] and driving anti-inflammatory cytokine production. This indicates its role as a modulator of inflammatory response in the presence of persistent bacterial load.

IL-35, a member of the IL-12 family, is an anti-inflammatory cytokine responsible for immune system maintenance, leading to Treg expansion and Th17 cell inhibition [37]. IL-35 is not constitutively expressed in human tissue, but it is produced in response to inflammatory stimuli [38], such as periodontitis. In accordance to Kalburgi et al. [39], IL-35 levels in our PD group were significantly higher than those of the healthy subjects, with a reduction in stage IV compared to stages II and III, suggesting a regulatory role in periodontitis pathogenesis.

IL-17A is secreted by Th17 cells and is responsible for amplifying the innate immune response by regulation of receptor and chemokine expression. In our study, proinflammatory IL-17A also seemed to be upregulated in the saliva of the PD group than in the controls. During PD, alveolar bone loss is driven by the increase of IL-17, reduction of Treg cytokines, and Th17/Treg ratio imbalance, leading to an increased inflammatory state, characteristic of chronic periodontitis [7].

MMP2 and MMP9 are essential for the initiation of bone remodeling, responsible for managing acute and chronic inflammatory responses [40,41]. In the present study, an increase in MMP9 levels was detected in periodontopathic patients rather than in healthy subjects. This is in accordance with different studies [42,43] that have reported matrix metalloproteinases as widely accepted disease markers due to the role of collagen as a major structural protein in the periodontium.

Overall, our results point to a pivotal role of TGFβ in periodontitis. In fact, in our patients, it was positively correlated with both pro- and anti-inflammatory cytokines (IL-17A, IL-35; rho = 0.7), confirming its regulatory role in immune response. Furthermore, in the relationship between TGFβ and MMP9, weak correlation was observed, which may support recent studies demonstrating that MMP9 is a downstream mediator of TGFβ that inhibits cell invasion and metastasis in TGFβ-induced cells [44].

Moreover, in healthy subjects, a similar trend of positive correlation between TGFβ and the other cytokines was observed, assuming its key role in preventing disease development. The positive correlation between TGFβ and MMP9 confirmed the multifunctional activity of both molecules involved in the protection and pathogenesis of oral cavity diseases. In accordance with Li et al., a weak relationship between TGFβ and IL-35 in healthy controls was detected due to a lack of IL-35 cytokine expression in the absence of chronic inflammatory stimulus [38].

Salivary components (i.e., cytokines, vitamins) may derive from both blood and salivary glands, by passive diffusion or active transport, without significant interference from the salivary gland alterations. Therefore, cytokines and vitamins present in saliva can mirror blood levels, making saliva an effective fluid for biomarker research [45,46].

In our PD group, salivary 25(OH)D_3_ was inversely linked to TGFβ (*p* < 0.05), IL-35 (*p* < 0.05), and IL-17A (*p* < 0.05) levels with statistical significance. Different studies have shown that vitamin D and TGFβ follow different signal pathways, and VDR receptor activation can be considered as a negative regulator of TGFβ signaling [47,48,49]. TGFβ has been reported to reduce dendritic cell (DC) differentiation, while vitamin D downregulates Th1 activity. Significant negative correlation between 25(OH)D_3_ levels and IL-35 was observed in our patients, suggesting that IL-35, a Treg suppressor, could be responsible for inflammatory response balance in the presence of bacterial infection, limiting immune system overactivation [50].

Confirming the data of Oh et al. [51], which showed that vitamin D downregulates MMP9 production in the presence of hypovitaminosis by blocking NFkB pathway activation, the increase of MMP9 in our PD patients is plausible. In relation to disease stage, MMP9 followed an increase (*p* < 0.05) in the presence of increased periodontal pocket depth, favoring tooth loss and the establishment of chronic inflammation [52].

The role of vitamin D on Th17 cell activity has been evaluated in several diseases, and it has been shown that vitamin D suppresses IL-17 and IL-23 expression by reducing retinoic-acid-related orphan receptor γt expression [53]. Proinflammatory IL-17 production was increased in our patients, in accordance with other studies showing the key activity of the 1,25(OH)2D/VDR signaling pathway in fine-tuned immune homeostasis maintenance through the negative modulation of proinflammatory cytokines IL-2, IL-17, and IFNα [7,54].

Similar results were obtained for our HC, showing significant inverse correlation between vitamin D and TGFβ (*p* < 0.05) and IL-17A (*p* > 0.05). Cytokines are critical mediators of immune responses, and their relationship with vitamin D is yet to be elucidated. As recently reported by Meghil et al. [55], vitamin D supplementation can re-establish the optimal vitamin D levels, leading to multiple benefits for reducing local and systemic inflammation.

Moreover, salivary cytokines are important to evaluate host susceptibility, which represent a factor for periodontal disease pathogenesis, in relation to microbial stimulation and cytokine inflammatory pathways [56].

Keeping this in mind, our results, albeit preliminary, showing significantly different levels of cytokine in saliva of PD patients in comparison with healthy controls, suggest that saliva, alone or in association with other molecules, such as vitamin D, might have the potential to act as novel diagnostic markers for periodontitis.

In summary, this study is one of the first to simultaneously evaluate the salivary levels of cytokines and 25(OH)D_3_ in patients with periodontal disease. Our results showed high cytokine levels and lower vitamin D levels in PD patients compared to healthy controls. The increase of TGFβ salivary level, associated with worse periodontal conditions and with pro-/anti-inflammatory molecules, leads us to speculate that TGFβ plays an important role in PD.

The major limitation of this study is the small sample size. Further well-organized studies with large sample size and more sophisticated analytic techniques are needed to give additional information and to establish the use of cytokines and vitamin D salivary levels in clinical practice for the prediction of periodontal progression and stability. Within the limits of the study, our results support the hypothesis that low levels of 25(OH)D_3_ is related to inflammatory response, increasing the secretion of anti-inflammatory cytokines. Thus, it could be speculated that vitamin D assessment, and its possible implementation in deficiency cases, could play a role in periodontal treatment.

## 4. Materials and Methods

### 4.1. Population

The sample population included 42 male and female subjects aged from 25 to 60 years old. The enrolled subjects were patients from the dental clinic of the Department of Medical, Oral, and Biotechnological Sciences, G. d’Annunzio University of Chieti–Pescara, Italy.

Inclusion criteria for the healthy controls were as follows: no systemic diseases, no medications affecting periodontal status during the previous 6 months, not pregnant or lactating, a FMPS and a FMBS ≤20%; no diagnosis of periodontitis in the past or present. Healthy controls were patients who had gingival index <1 mm, PPD <3 mm, and no CAL. Meanwhile, for the test group, the inclusion criteria were as follows: patient with a diagnosis of periodontitis done according to the new classification of periodontal disease [57] and had a minimum of two teeth with probing pocket depth (PPD) ≥4 mm, CAL ≥3 mm, and positive bleeding on probing in involved areas.

Exclusion criteria for all recruited subjects were as follows: presence of any chronic disease (kidney, intestinal, liver, heart, etc.); hypertension; autoimmune inflammatory disease or diabetes; systemic bacterial, viral, or fungal infection; diagnosis of osteoporosis or osteopenia treatment within the past six months with antibiotics, anti-inflammatory, osteoporosis treatment, bisphosphonates, or multivitamin supplements; and periodontal treatment received for the past year. Moreover, all patients were screened for salivary gland disease, Sjögren syndrome, and oral cancer.

Participants answered a questionnaire containing data about age, sex, daily habits, hormonal, and vitamin D status. After inclusion in the present study, patients were divided into two groups: healthy controls (HC; *n* = 21) and periodontal disease patients (PD; *n* = 21). All patients gave their written informed consent before beginning the study.

The participants who fulfilled the requirement of generalized chronic periodontitis in the PD group were divided into three subgroups in accordance with the new classification of periodontal disease as described in detail below by the 2017 World Workshop. Specifically, they were allocated in Chronic Periodontitis group II, Chronic Periodontitis III, and Chronic Periodontitis IV based on probing depth, CAL, FMPS, FMBS, and radiographic bone loss. Periodontopathic patients received nonsurgical comprehensive periodontal treatment (full-mouth scaling, root planning, polishing, and individualized oral hygiene instruction) after the periodontal examination. PD and HC features are shown in detail in Table 1 and Table 4. The study was approved on 14 November 2019 by the Interinstitutional Ethics Committee of the University of Chieti–Pescara, Chieti, Italy; committee report no. 254.

### 4.2. Clinical Periodontal Parameter Examination

Diagnosis and classification of periodontal disease were done according to the new classification of periodontal disease, defined in the context of the 2017 World Workshop on the basis of clinical and radiographic data. Periodontitis is characterized by microbially associated host-mediated inflammation that results in periodontal attachment loss. Conventional clinical periodontal parameter measurements, such as CAL and maximal probing depth, are used for diagnosis of previous periodontal disease rather than present disease activity. CAL was measured by circumferential assessment of the erupted dentition with a standardized periodontal probe concerning the cementoenamel junction (CEJ). This classification is based on stage and grade to appropriately define periodontitis in an individual patient. Stages I–IV of periodontitis are defined on the basis of severity (primarily periodontal breakdown regarding root length and periodontitis-associated tooth loss), management complexity (pocket depth, infrabone defects, furcation involvement, tooth hypermobility, masticatory dysfunction), and extent (localized or generalized) [57]. Clinical examination of periodontal disease was performed at the periodontology department, and the stage of periodontal disease was defined by carrying out a six-point probing for each element performed with a North Carolina PCP15 probe. Full-mouth intraoral X-ray series of patients’ teeth and adjacent hard tissues were taken. However, in this evaluation, only periapical X-rays and no bitewing X-rays were performed. The periapical radiographs were taken by the paralleling technique using a Kodak Ultraspeed (Carestream Health, Milan, Italy). Probing was performed with vertical movements and without ever leaving the depth, performing it on six sites (three vestibular and three lingual/palatine) for each tooth. In this way, the probe was kept parallel to the long axis of the tooth to avoid detection errors. For this clinical patient evaluation, the stage, but not grade, related to prognosis establishment was taken into consideration.

### 4.3. Saliva Sample Collection

After clinical periodontal parameter examination, whole unstimulated saliva samples were collected from each patient. About 5 mL of unstimulated whole saliva was collected from each subject in sterile polypropylene tubes. Samples were centrifuged at 10,000× *g* for 15 min at 4 °C, and the supernatant was immediately aliquoted and frozen (−80 °C) until analysis. Biosafety level 2 procedures were applied for working with patients’ samples.

### 4.4. Vitamin D and Cytokine Measurement

Levels of 25(OH)D_3_ (Cayman Chemical, Michigan, USA), TGFβ (Boster, Pleasanton, CA, USA), IL-35 (Elabscience, Houston, USA), IL-17A (Diaclone, Besancon, FR), and MMP9 (BioVendor, Brno, CZ) were assayed by the ELISA technique using a manufacturer kit. The limits of detection were 25(OH)D_3_ and MMP9, <0.5 ng/mL; TGFβ, IL-35, and IL-17A ≤ 1 pg/mL. For all assays, absorbance was assessed using a Glomax multidetection reader spectrophotometer (Promega, Milan, Italy).

### 4.5. Statistical Analysis

Descriptive statistics relied on median and IQR for quantitative variables and on frequencies and percentages (%) for qualitative variables. Before carrying out nonparametric analysis, normality was checked using the Shapiro–Wilks test. To evaluate the relationship between numerical variables, we used the Spearman rank correlation coefficient; the polyserial correlation coefficient was used to assess the relationship between ordinal and numerical variables. For all tests, the threshold for statistical significance was set at *p* = 0.05. All analyses were performed with open-source statistical R software (version 3.4.3; R Foundation for Statistical Computing).

## Figures and Tables

**Table 1:** Periodontal parameters.

Parameters	Disease Stage		
	II (*n* = 7)	III (*n* = 9)	IV (*n* = 5)
Maximum probing depth (mm)	5 (4–5)	7 (6–9)	7 (6–7)
CAL 12-21 (mm)	4 (3–4)	5 (5–5)	7 (5–7)
CAL 31–41 (mm)	4 (3–4)	5 (3.5–5)	7 (6–8)
CAL 36 (mm)	4 (3–4)	6 (5–7)	6 (6–8)
CAL 46 (mm)	4 (3–4)	6 (5–7)	6 (5–6)
CAL 26 (mm)	3 (3–4)	5 (5–6)	6 (6–6)
CAL 16 (mm)	4 (3–4)	5 (5–5)	5 (5–7)
FMBS (%)	41 (39–49)	48 (43–55)	48 (41–60)
FMpS (%)	36 (34–39)	48 (39–52)	53 (40–64)
Radiographic_bone_loss (%)	31 (28–32)	65 (44–70)	80 (75–83)

Data summarized as median and interquartile range (IQR). *p*-value derived from Kruskal–Wallis test. CAL: clinical attachment level; FMPS: full-mouth plaque score; FMBS: full-mouth bleeding score.

**Table 2:** Salivary mediator levels.

	HC Median (IQR)	PD Median (IQR)	*p*-Value
TGFβ (pg/mL)	34.8 (30.7–55.2)	156.9 (130.4–231.8)	<0.001
IL-35 (pg/mL)	43.5 (36.1–49.9)	98.37 (62.5–306.8)	<0.001
IL-17A (pg/mL)	8.3 (4.3–11.1)	21.3 (12.3–28.3)	<0.001
MMP9 (ng/mL)	198.0 (152.1–352.1)	388.9 (189.4–709.6)	0.209
25(OH)D_3_ (ng/mL)	4.6 (3.1–8)	3.7 (2.0–5.3)	0.207

Data summarized as median and interquartile range (IQR); *p*-value derived from Mann–Whitney *U* test. HC: healthy control; PD: periodontal disease.

**Table 3:** Salivary marker level correlation.

	25(OH)D_3_		TGFβ		IL-35		IL-17A	
	PD	HC	PD	HC	PD	HC	PD	HC
TGFβ (pg/mL)	−0.71 *	−0.63 *						
IL-35 (pg/mL)	−0.73 *	−0.24	0.71 *	−0.23				
IL-17A (pg/mL)	−0.71 *	−0.60 *	0.70 *	0.65 *	0.68	−0.12		
MMP9(ng/mL)	−0.10	−0.38	0.46	0.81 **	0.23	−0.37	−0.15	0.47

Data represent Spearman rho correlation coefficient obtained from salivary marker levels in PD group and in HC. Significance code, * *p* < 0.05; ** *p* < 0.01.

**Table 4:** Patient parameters.

	PD (*n* = 21)	HC (*n* = 21)	*p-*Value
Age (years), mean ± SD	56.9 ± 5.4	54.3 ± 5.0	0.113
Gender, *n* (%)			1.000
Male	7 (33)	7 (33)	
Female	14 (67)	14 (67)	
Hormonal status in female, *n* (%)			0.648
Fertile	2 (14)	4 (29)	
Menopause	12 (86)	10 (71)	
BMI (Kg/m^2^), *mean* ± *SD*	22.3 ± 2.6	24.5 ± 4.2	0.055
Current smoker, *n* (%)	2 (9)	3 (14)	0.999

Data summarized as mean ± standard deviation. *p*-value derived from Mann–Whitney *U* test for quantitative variables and chi-squared test for qualitative variables. PD, periodontopathic group; HC, control group; BMI: body mass index.

## Abbreviations

PD	Periodontal disease
HC	Healthy control
IL	Interleukin
TNF	Tumor necrosis factor
IFN	Interferon
Th	T helper
TGF	Transforming growth factor
MMP	Matrix metalloproteinase
CAL	Clinical attachment loss
CEJ	Cementoenamel junction
FMPS	Full-mouth plaque score
FMBS	Full-mouth bleeding score
IQR	Interquartile range
ECM	Extracellular matrix
NLRPL3	Nod-like receptor leucine-rich repeat protein 3
VDR	Vitamin D receptor
GCF	Gingival crevicular fluid
Treg	T regulatory cell
